# Single-cell sequencing analysis reveals gastric cancer microenvironment cells respond vastly different to oxidative stress

**DOI:** 10.1186/s12967-022-03411-w

**Published:** 2022-06-03

**Authors:** Weihua Yu, Guojun Chen, Jiafei Yan, Xianfa Wang, Yiping Zhu, Linghua Zhu

**Affiliations:** Department of General Surgery, Sir Run Run Shaw Hospital, Zhejiang University School of Medicine, 3 East Qingchun Road, Hangzhou, 310016 Zhejiang China

## Abstract

Gastric cancer is a common type of gastrointestinal malignant tumor in China. The mechanism of the development and progression of gastric cancer remains the continuing research focus. The tumor microenvironment plays an important role in the development and progression of tumors. The present study used single-cell sequencing data to characterize the microenvironment of gastric cancer, investigate the effects of oxidative stress on gastric cancer microenvironmental cells through the comparison between cancer tissue and normal tissue, and identify the key genes associated with gastric cancer patients’ survival. The results showed that compared with normal gastric tissue, gastric cancer tissue had a decreased oxidative stress response, weaker oxidative detoxification ability, and increased oxidative stress-induced cell death. In the different types of single cells of gastric cancer microenvironment, the oxidative stress response of T cell was increased, the ability of oxidative detoxification was enhanced, and the oxidative stress-induced cell death was exacerbate. Mucous cell showed the same trend as gastric cancer cells: decreased oxidative stress response, weak oxidative detoxification ability, and weakened oxidative stress-induced cell death. Moreover, TRIM62, MET, and HBA1, which were significantly associated with oxidative stress, may be biomarkers for the prognosis of gastric cancer. High expression of TRIM62 indicated a good prognosis, while MET and HBA1 indicated a poor prognosis, which will be confirmed by further clinical studies.

**Supplementary Information:**

The online version contains supplementary material available at 10.1186/s12967-022-03411-w.

## Introduction

Gastric cancer is the most common malignant tumor of the digestive tract and it is the fourth leading cause of cancer mortality worldwide [1]. The statistics from the International Agency for Research on Cancer in 2020 show that there were approximately 1.089 million new cases of gastric cancer and 769,000 deaths worldwide in that year, of which 43.9% of the cases and 48.6% of the deaths occurred in China [2]. At present, the overall treatment model for gastric cancer has undergone significant changes. It has evolved from anatomy-based surgical resection treatment to a comprehensive treatment model based on anatomy, tumor biology, and immunology [3]. The immune clinical treatment of gastric cancer still faces great challenges, which has a certain connection with the microenvironment of gastric cancer.

The microenvironment of gastric cancer refers to the internal environment in which gastric cancer occurs and develops. It is composed of gastric cancer cells, microvessels, microlymphatic vessels, interstitial cells, tissue fluid, immune and inflammatory cells, numerous cytokines, and a small amount of infiltrating cells, etc. [4]. The microenvironment of gastric cancer is quite complex, and it is very different from the microenvironment of normal tissues; among them, inflammation is the key initial step in the occurrence of gastric cancer, and it is the main origin of gastrointestinal cancer [5]. Many inflammatory factors, such as TNF-α, IL-6, TGF-β, and IL-10, have been confirmed to be involved in the occurrence and development of tumors. In this process, the levels of reactive oxygen and nitrogen are affected, and the oxidative stress caused by them is the main cause of deoxynucleotide damage and tumor transformation [6]. The interaction between the tumor and its microenvironment is also quite complex. Exposure of cancer stem cells to the microenvironment of reactive oxygen can stimulate the antioxidant system to enhance the antioxidant capacity and acquire the malignant phenotype [7].

Oxidative stress refers to a state of imbalance between oxidation and antioxidant effects in the body. Kruk, et al. summarized the carcinogenic effects of reactive oxygen and nitrogen. Oxidative stress is not only a major cause of gastrointestinal mucosal diseases but also relevant in the occurrence and development of gastric cancer [8]. This study will combine The Cancer Genome Atlas (TCGA) gastric cancer sequencing data and gastric cancer single-cell sequencing data to describe the gastric cancer microenvironment. Through the comparison between pathological tissues and normal tissues, analyze how oxidative stress affects microenvironmental cells, and discover the risk genes that have a key impact on the survival of gastric cancer patients.

## Materials and methods

### Data collection and processing

We downloaded and collected the data of gastric cancer patients in the TCGA project from the UCSC Xena (https://xenabrowser.net/datapages/) database, including RNA-sequencing analysis of gene expression profiles and patient clinical information. We also collected and downloaded all GO: BP (GO BP terms; biological process GO terms) from the Molecular Signatures Database (MsigDB; https://www.gsea-msigdb.org/gsea/msigdb) for the evaluation of oxidative activation related functions. In addition, we chose to collect and download a set of gastric cancer-related single-cell sequencing data, GSE112302, from the Gene-Expression Omnibus (GEO; https://www.ncbi.nlm.nih.gov/geo/) database.

### ssGSEA algorithm calculates the function score

To assess differences in oxidative stress-related functions in samples, we downloaded GO BP terms in the MsigDB database to select the required function items, then used the R package "GSVA" to score each sample with the single sample GSEA (ssGSEA) algorithm. The higher the score, the higher the relative level of gene expression related to the functional item of each sample. We use this to evaluate the activation state of each sample in the functional item [9].

### Quality control and processing of single cell data

For the GSE112302 single-cell sequencing data obtained from the GEO database, we have obtained the Transcripts Per Million (TPM) expression matrix of every single cell and performed quality control. We counted the number of genes expressed in every single cell and the number of cells expressed by each gene, determine the quality control threshold according to the overall distribution and eliminate low-quality single cells and genes. After the preliminary quality control, the R package "Seurat" was used to carry out a standardized analysis process. We first used "NormalizeData" to standardize the data, then used "RunPCA" for principal component analysis. Finally, single-cell samples were clustered using the k-nearest neighbor classification (KNN) algorithm through "FindNeighbors" and "FindClusters" [10].

### Prognostic analysis

To assess the impact of genes on the prognosis of gastric cancer patients, we combined the prognostic survival clinical information of gastric cancer patients in the TCGA database, used the R package "survival" to construct a Cox proportional hazard model and performed a log-rank test, and recorded the *HR (*95%*CI*) and the *P*-value of the survival model.

### Screening and functional annotation of differentially expressed genes

To explore the differences in the expression of cells derived from normal tissues and tumor tissues, we separately counted the average expression of each gene in normal cells and tumor cells, calculated the Fold Change value, and recorded the *P*-value of the Wilcoxon rank-sum test. The screening threshold was |log2FC|> 1 and *P*-value is < 0.01. In addition, we also use the DAVID function to perform functional annotations in response to differentially expressed genes.

### Statistical analysis

The following statistical models and methods were used in this study: (1) Wilcoxon rank-sum test is used to compare the difference between two sets of data; (2) The Cox proportional hazard model is used to evaluate the impact of gene expression levels on the prognosis of gastric cancer patients; (3) Log-rank test is used to assess whether gene expression levels significantly affect patient survival.

## Results

### Oxidative stress is different in normal and gastric cancer tissues

The human body is always in a state of balance between the oxidation system and the antioxidant system. Oxidative stress refers to the pathological process in which the body produces too much active oxygen or the antioxidant capacity is reduced, and the oxidative system and the antioxidant system are out of balance, which leads to the pathological process of potential damage. The response to oxidative stress is the basis for ensuring the normal metabolic activity of cells. Therefore, we downloaded the gastric cancer tissue RNA-sequencing sample data from the TCGA database and compared the response of normal gastric tissue and gastric cancer tissue under oxidative stress.

**Fig. 1:**
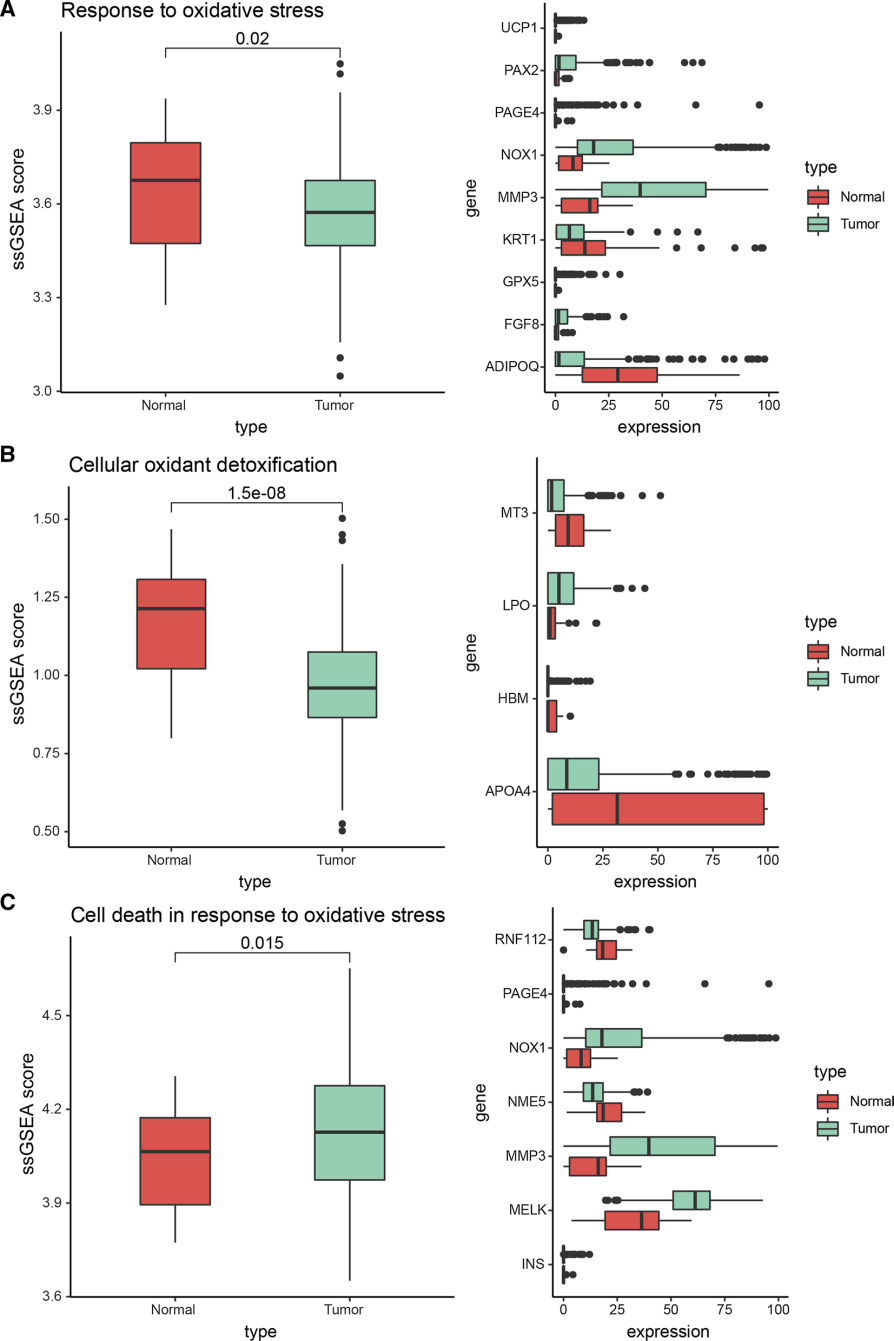
**A** Difference of cellular oxidative stress response in TCGA gastric cancer samples. **B** Differences in the detoxification ability of cellular oxidants in TCGA gastric cancer samples. **C** Differences in the ability of oxidative stress-induced cell death in TCGA gastric cancer samples, the picture on the left shows the ssGSEA scores for specific GO BP terms in the two types of samples; The higher the score, the more active the function is. The figure on the right shows the expression of differentially expressed genes in the two types of samples in this term

According to the relevant functional items collected from the MsigDB database, we used the ssGSEA algorithm to score each TCGA gastric cancer sample for oxidative stress response, including 35 normal gastric tissue samples and 415 gastric cancer samples. First, we evaluated the "GO_RESPONSE_TO_OXIDATIVE_STRESS" of the two types of samples. We found that the response level of gastric cancer samples to oxidative stress was generally lower than that of normal gastric tissue samples (*P* = 0.02) (Fig. 1A). We further evaluated the “GO_CELLULAR_OXIDANT_DETOXIFICATION” cell oxidation and detoxification capabilities of the two types of samples and found that the oxidation and detoxification capabilities of gastric cancer samples were generally weaker than that of normal gastric tissue samples (*P* = 1.5e−8) (Fig. 1B).

Finally, we also evaluated the functional scores of "GO_CELL_DEATH_IN_RESPONSE_TO_OXIDATIVE_STRESS" for oxidative stress-induced cell death in two types of samples, and found that the ssGSEA score of tumor samples was significantly higher than that of normal gastric tissue samples (*P* = 0.015) (Fig. 1C).

### Oxidative stress response and microsatellite instability

**Fig. 2:**
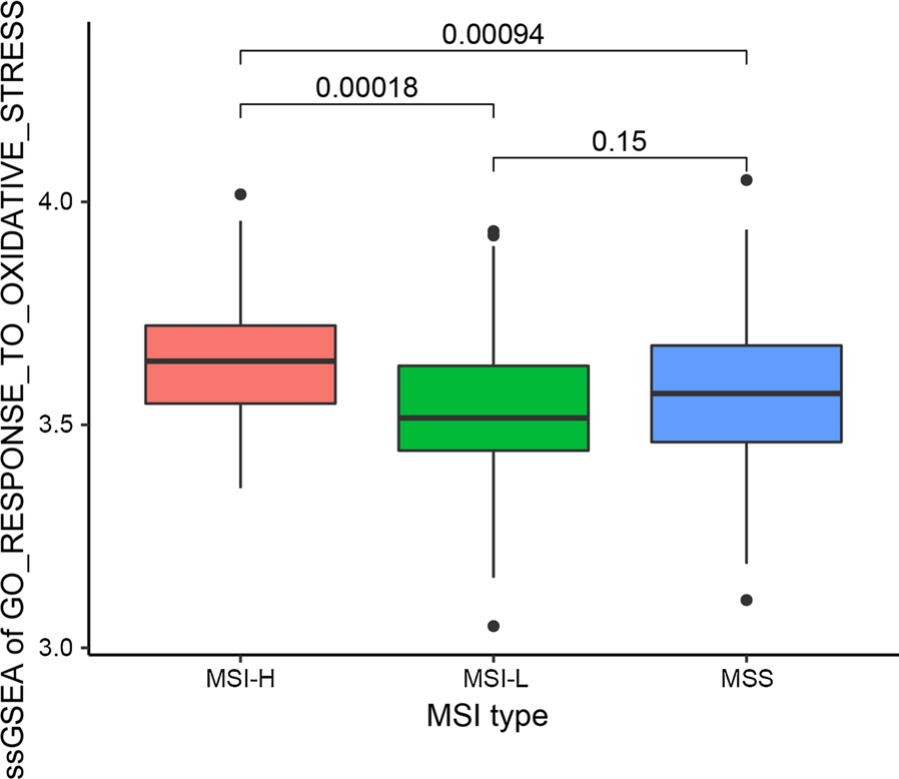
SsGSEA score and microsatellite instability in response to oxidative stress

Oxidative stress can cause damage to cell DNA or RNA. Microsatellite instability (MSI) is one of the indicators of chromosome instability. We suspect that there will be a certain relationship between the two. Therefore, we collected and sorted out the MSI of 450 gastric cancer patients in the TCGA database (Table 1). According to the MSI, gastric cancer patients are mainly divided into three categories: MSI, MSI-L and MSI-H. We also use the "ssGSEA" algorithm to calculate the "GO_RESPONSE_TO_OXIDATIVE_STRESS" oxidative stress response ssGSEA score of the three types of gastric cancer patients. We found that MSI-H gastric cancer patients had the most active oxidative stress response (MSI-H *vs.* MSI: *P* = 0.00094; MSI-H *vs.* MSI-L: *P* = 0.00018), but there is little difference between MSI-L and MSS gastric cancer patients (*P* = 0.15) (Fig. 2). The above results indicated that the tumor tissue of patients with MSI-H STAD has an active oxidative stress function, which causes DNA and RNA damage, and ultimately leads to chromosomal instability.

**Table 1:** Results of microsatellite instability in gastric cancer patients

MSI type	Patients number
MSI	296
MSI-L	67
MSI-H	86
Unknown	1

### Gastric cancer microenvironment ecology

In the above analysis, we found that gastric cancer tissue is relatively inactive in response to oxidative stress, and its ability to oxidize and detoxify is relatively weak, while oxidative stress-induced cell death is strengthened. But considering that this is not consistent with the phenomenon of tumor cells evading cell death. The analysis is too consistent. The possible reason is that the above analysis is carried out in tissue samples, and the tissue samples are mixed with tumor cells and microenvironmental cells. Therefore, we also downloaded a set of single-cell sequencing data of gastric cancer from the GEO database, GSE112302, to distinguish between tumor cells and microenvironmental cells for analysis. The single cells were derived from 6 tumor tissues and 4 normal tissues. A total of 707 single cells were detected and 24,135 genes were captured.

**Fig. 3:**
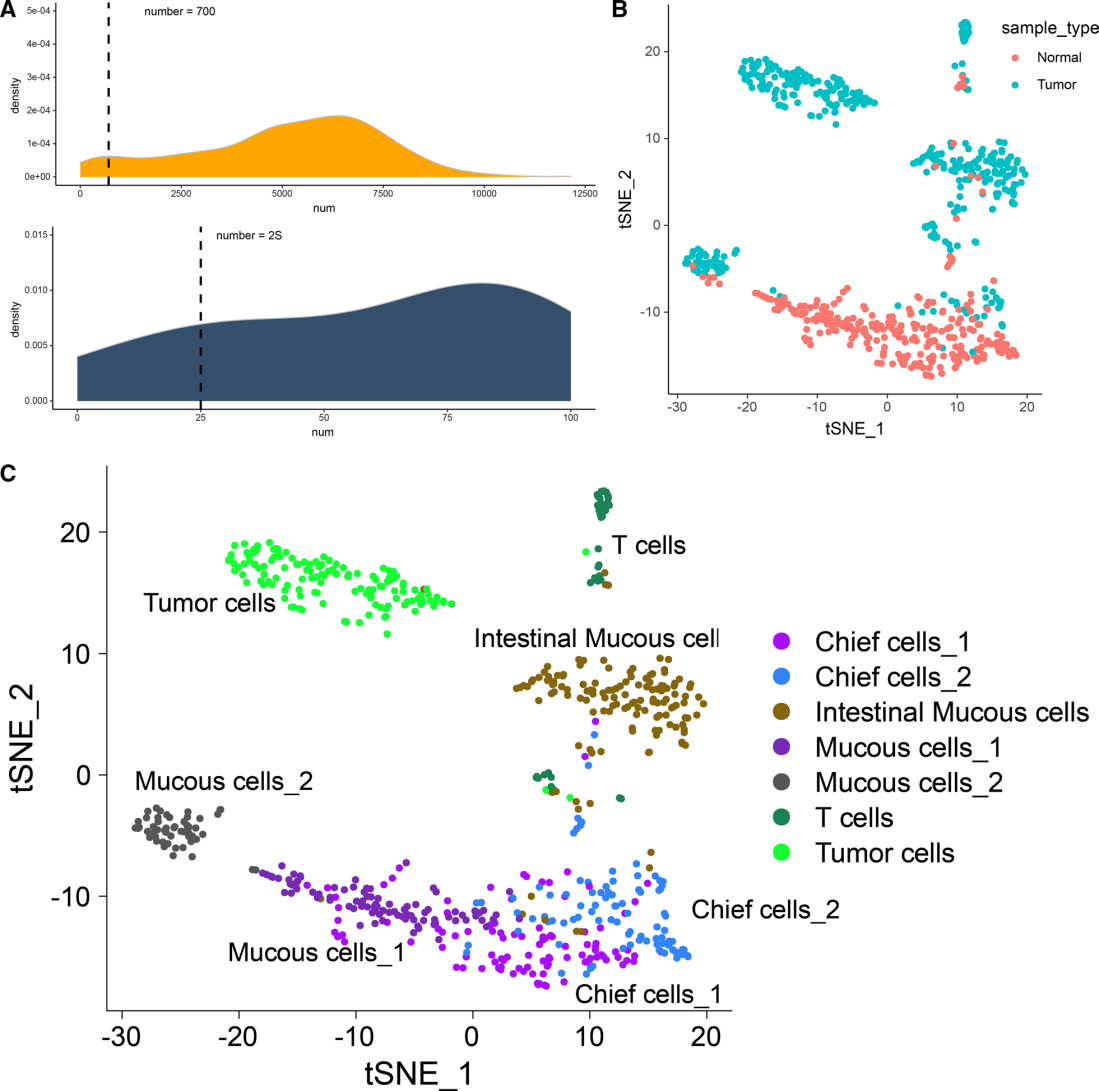
**A** Single cell quality control chart; the figure above counts the number of genes expressed in each single cell, and it is considered that cells expressing less than 700 genes are low-quality cells; the figure below counts the number of cells expressing each gene. It is considered that genes expressed in less than 25 cells are low-quality genes. **B** TSNE chart shows the sample source of the single cell. **C** TSNE chart shows the cell type of the single cell

First of all, we follow the part described in 2.3, preliminary quality control has been carried out to eliminate low-quality genes and cells. We counted the number of genes expressions in each single cell, divided the threshold according to the overall distribution to 700, and eliminated cells with less than 700 expressed genes (Fig. 3A). We also counted the number of cells expressed for each gene, divided the threshold according to the overall distribution to 25, and the genes expressed in less than 25 cells are eliminated (Fig. 3B). At this time, the expression profile we got includes 663 cells and 14,086 genes.

**Fig. 4:**
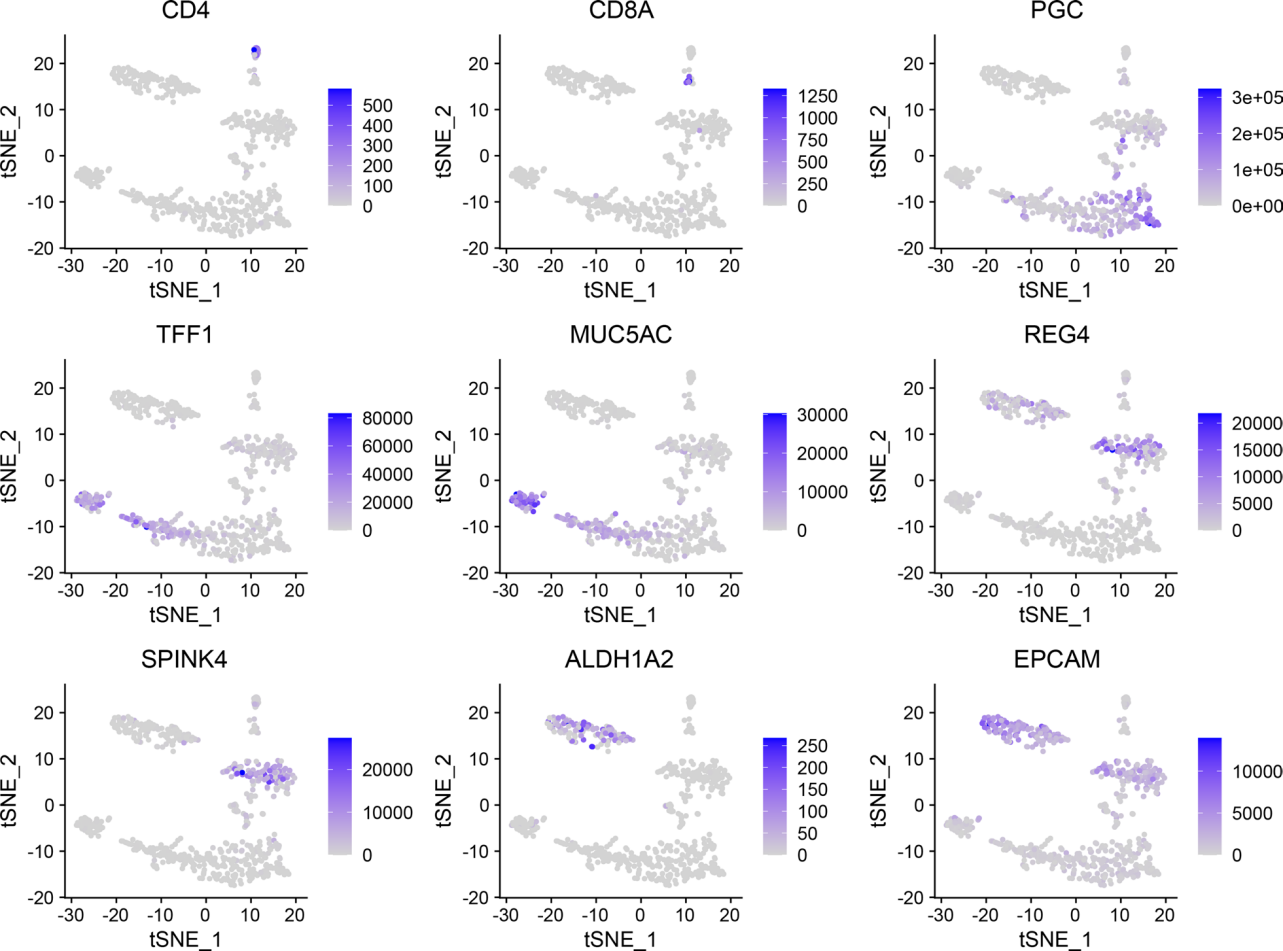
The specific expression marker in stomach cells

Subsequently, we used the R package "Seurat" to standardize single cells, including data standardization, PCA dimensionality reduction, KNN, etc. In the end, we obtained 7 cell subgroups (Table 2, Fig. 3C). According to the signature expression markers of stomach cells (Fig. 4), we identified CD4 + T cells, CD8 + T cells, mucous cells (TFF1 + and MUC5AC +), intestinal mucous cells [REG4 + and SPINK4 +], Chief cells (PGC +), and the potential gastric cancer cells (ALDH1A2 + and EPCAM +) completely derived from gastric cancer tissues. Among them, ALDH1A2 is a symbolic marker of ovarian cancer cancer stem cell, and it may also be a potential marker of gastric cancer stem cells.

**Table 2:** Single cell statistics of normal and gastric cancer tissues

Cell type	Normal tissue	tumor tissue
Intestinal mucous cells	11	128
Chief cells_1	97	9
Mucous cells_1	87	2
Chief cells_2	69	20
Mucous cells_2	8	45
T cells	9	40
Tumor cells	0	138

### Single cell oxidative stress in gastric cancer

Furthermore, based on the analysis results in tissue samples, we believe that in the tumor microenvironment, tumor cells and cells in the microenvironment should have different responses to oxidative stress. Therefore, we also used the "ssGSEA" algorithm in single-cell samples to calculate the "GO_RESPONSE_TO_OXIDATIVE_STRESS" oxidative stress response ssGSEA score for each single cell.

**Fig. 5:**
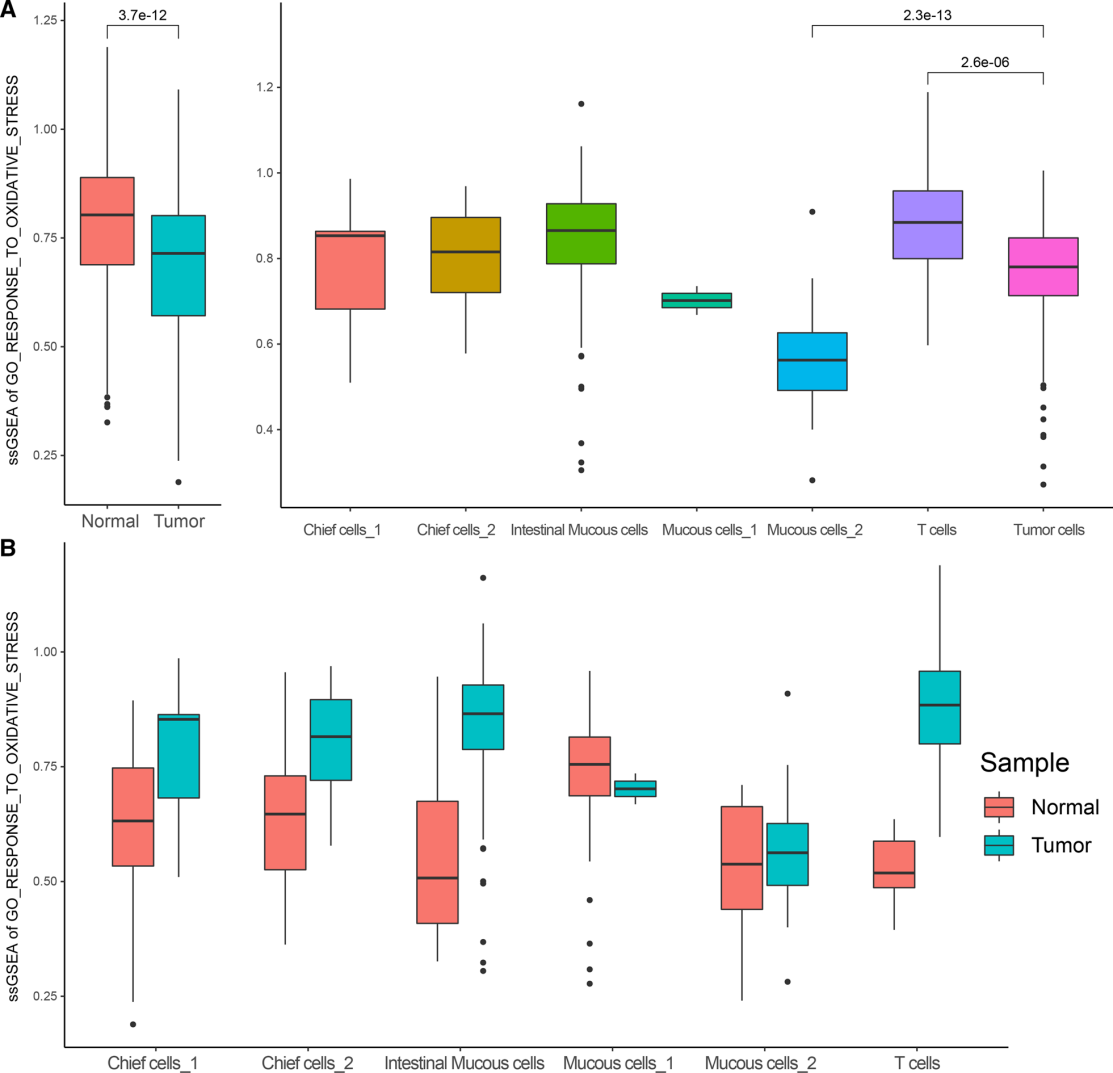
**A** Comparison of differences in oxidative stress response function scores in single cells; Note: The picture on the left is the comparison between all single cells derived from normal gastric tissue samples and single cells derived from tumor samples. On the right is a comparison between different cell populations of single cells derived from tumor tissues. **B** Difference of oxidative stress response function scores of microenvironmental cells in normal and tumor tissues

We found that single cells in gastric cancer tissues were the same as the results in tissue samples, and the oxidative stress response ssGSEA score was significantly lower than that of single cells in normal tissues (*P* = 1.9e−11). Moreover, when subdivided into different cell types of single cells in gastric cancer tissue, we found that the oxidative stress response ssGSEA score of gastric cancer cells in gastric cancer tissue is much lower than that of cells in the microenvironment. Among them, the oxidative stress of T cells is the most active (*vs.* gastric cancer cells, *P* < 2.2e−16); gastric cancer cells were also significantly lower than Mucous cells in the microenvironment that had the lowest ssGSEA score in response to oxidative stress (*P* = 0.012) (Fig. 5A).

Due to the observation that T cells in gastric cancer tissues are highly active in response to oxidative stress, we compared cell types derived from normal tissues and gastric cancer tissues (Table 3). We found that the ssGSEA score of T cells in gastric cancer tissues in response to oxidative stress was much higher than that in normal tissues, indicating that T cells in gastric cancer tissues were greatly affected by reactive oxygen (*P* = 0.001082013). Similarly, Chief cells_2 in gastric cancer tissues were also much higher than normal tissues (*P* = 5.46E−06). The Mucous cell_1 in the tumor tissue is lower than that in the normal tissue. This scoring trend is consistent with that of the tumor cells, indicating that the mucous cell in the tumor tissue has begun to become cancerous (Fig. 5B, Table 4).

**Table 3:** Differences in ssGSEA scores of microenvironmental cells in normal and gastric cancer tissues in response to oxidative stress

Cell type	Mean ssGSEA score in normal tissue	Mean ssGSEA score in tumor tissue	wilcox.test pValue
Intestinal mucous cells	0.554215362	0.839707069	7.50E−05
Chief cells_1	0.610178443	0.794530252	0.002211603
Mucous cells_1	0.741612364	0.701848027	0.325752726
Chief cells_2	0.62845311	0.803948135	5.46E−06
Mucous cells_2	0.527519743	0.567094677	0.651787564
T cells	0.525579952	0.878920957	1.17E−08

**Table 4:** Differences in ssGSEA scores of microenvironmental cells between normal and tumor tissues

Cell type	Mean ssGSEA score in normal tissue	Mean ssGSEA score in tumor tissue	wilcox.test pValue
Intestinal mucous cells	0.541419445	0.559288808	0.670670527
Chief cells_1	0.600140158	0.64749224	0.578636994
Mucous cells_1	0.599315732	0.508241537	0.299238166
Chief cells_2	0.597834707	0.649053443	0.059789554
Mucous cells_2	0.469452028	0.414158696	0.214021351
T cells	0.507775234	0.700627593	0.001082013

### Oxidation and detoxification ability of gastric cancer single cell

**Fig. 6:**
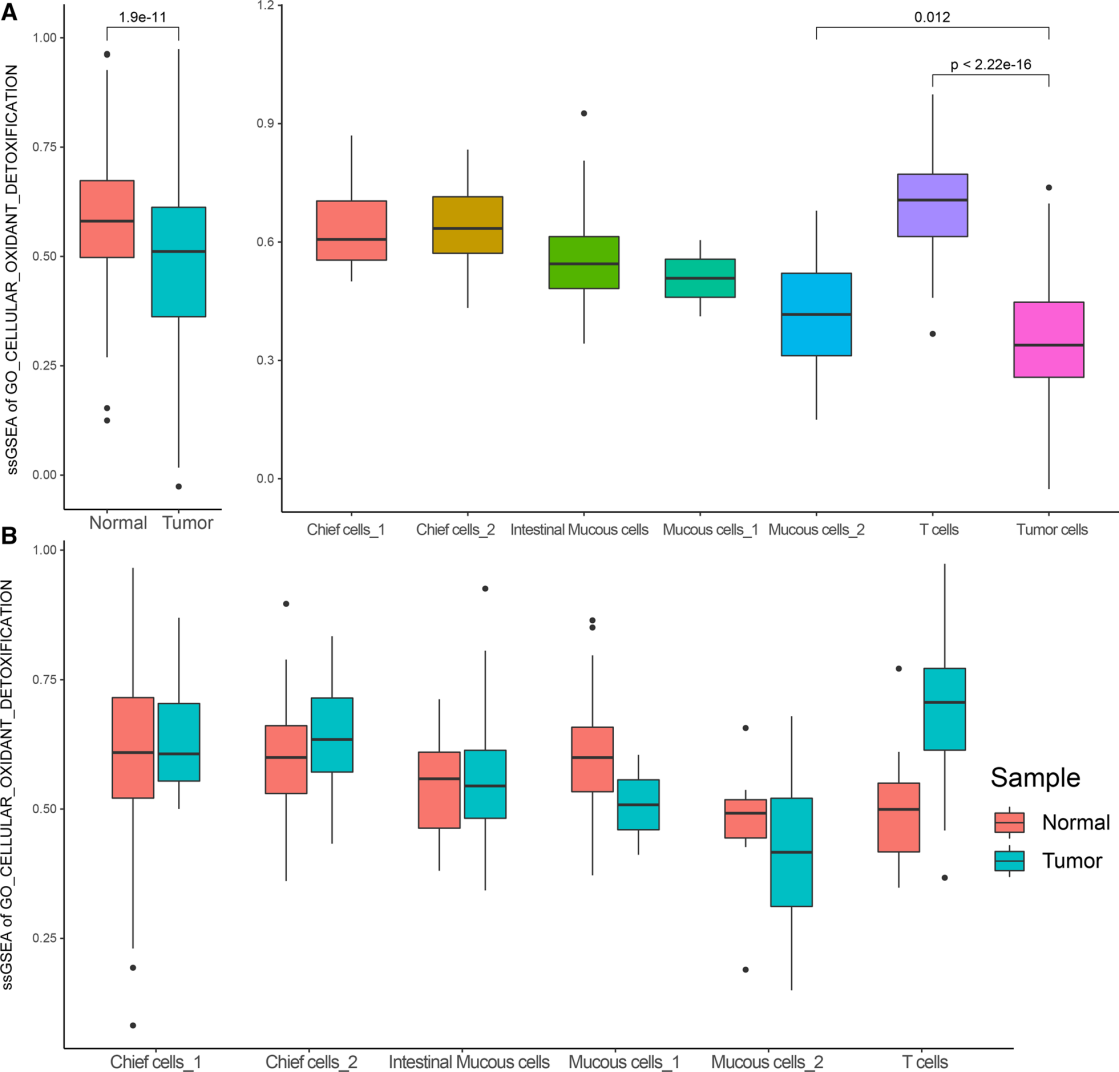
**A** Comparison of the difference of oxidative and detoxification function scores in single cells. **B** Difference of oxidation and detoxification scores of microenvironmental cells in normal and tumor tissues

We also carried out the analysis of the function score of "GO_CELLULAR_OXIDANT_DETOXIFICATION" oxidation and detoxification at the single cell level. We found that when specific to different cell types, the scores of other cell types except gastric cancer cells and mucous cells are higher, which further indicates that mucous cells may be a gradually cancerous cell (Fig. 6A).

In addition, we compared the tumor source and the normal tissue source according to the cell type, and found that the cells in the tumor microenvironment had a higher score for oxidation and detoxification of ssGSEA, especially T cells. Compared with normal tissue sources, T cells in gastric cancer tissues have significantly stronger oxidative and detoxification capabilities (*P* = 0.001082013) (Fig. 6B, Table 5).

**Table 5:** Difference of ssGSEA scores between normal and gastric cancer tissues in microenvironmental cells due to oxidative stress-induced cell death;

Cell type	Mean ssGSEA score in normal tiss	Mean ssGSEA score in tumor tiss	wilcox.test pValue
Intestinal mucous cells	0.554215362	0.839707069	7.50E−05
Chief cells_1	0.610178443	0.794530252	0.002211603
Mucous cells_1	0.741612364	0.701848027	0.325752726
Chief cells_2	0.62845311	0.803948135	5.46E−06
Mucous cells_2	0.527519743	0.567094677	0.651787564
T cells	0.525579952	0.878920957	1.17E−08

### Oxidative stress induced gastric cancer single cells death

Similarly, in the comparison of tissue samples, we found that the ssGSEA score of "GO_CELL_DEATH_IN_RESPONSE_TO_OXIDATIVE_STRESS" in gastric cancer tissues that oxidative stress-induced cell death was significantly higher than that of normal tissues. This phenomenon is contrary to the phenomenon of tumor cells evading cell death, so we also performed a single-cell level analysis.

**Fig. 7:**
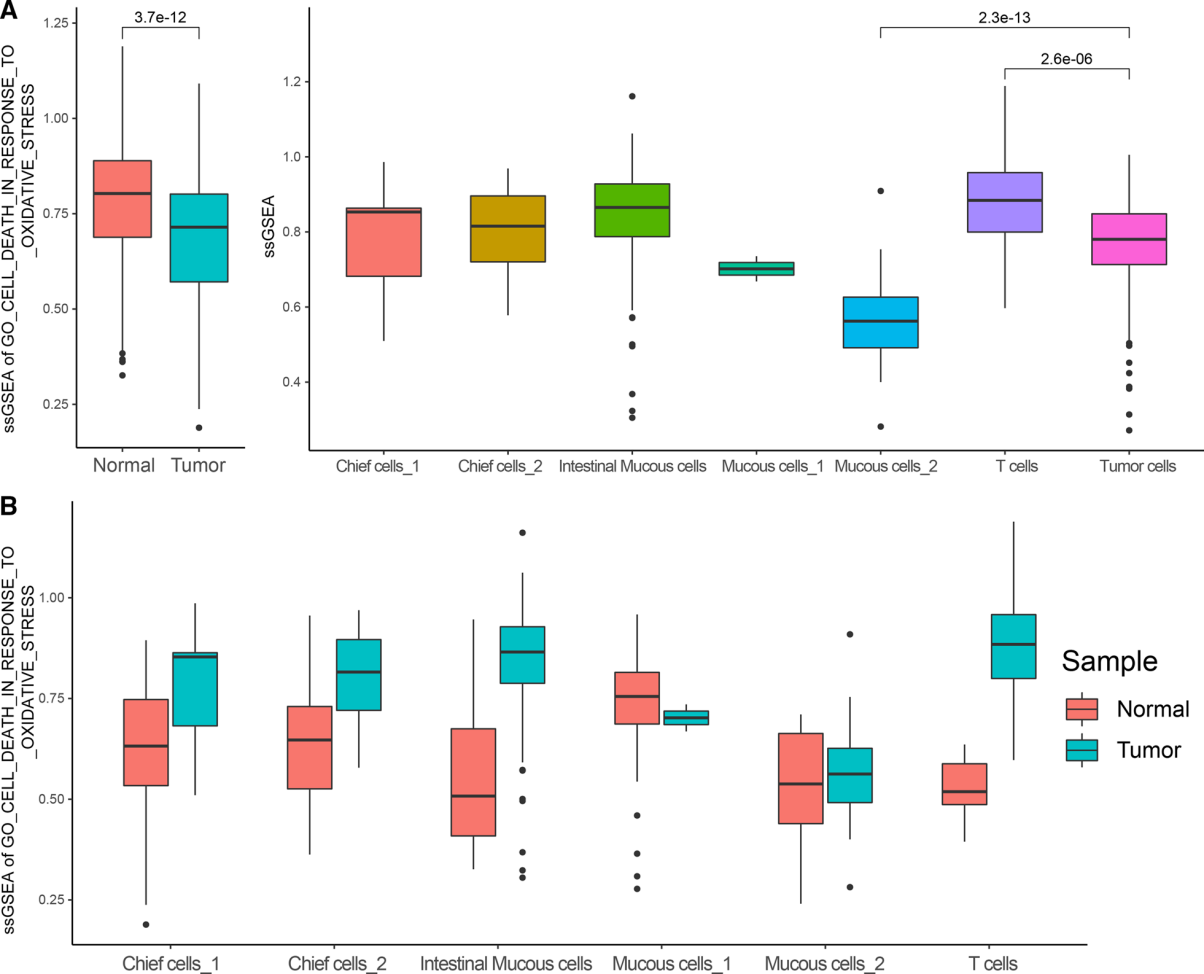
**A** Comparison of the difference in scores of cell death function caused by oxidative stress in single cells. **B** Difference in the scores of oxidative stress-induced cell death in the microenvironmental cells that release free calcium ions to the cytoplasm between normal and tumor tissues

The single-cell level results show that the single cells derived from gastric cancer are significantly lower (*P* = 3.7e−12). When specific to different cell types, other cell types except gastric cancer cells and Mucous cells have higher ssGSEA scores, which further indicates that Mucous cells may be a kind of gradually cancerous cell (Fig. 7A). In addition, we compared the levels of single cells derived from gastric cancer and normal tissues according to the cell type, and found that cells in the microenvironment of gastric cancer had higher ssGSEA scores for oxidative stress-induced cell death. It shows that cells in the microenvironment of gastric cancer tissues are all prone to cell death due to oxidative stress (Fig. 7B, Table 6).

**Table 6:** Functional annotation information of the top 200 genes in the tumor stem cell population;

Term	Count	PValue	Genes
GO:0006730 ~ one-carbon metabolic process	4	0.002955618	ALDH1L1, MAT2A, SHMT2, CA13
GO:0010719 ~ negative regulation of epithelial to mesenchymal transition	3	0.018828614	TRIM62, HPN, PBLD
GO:0032474 ~ otolith morphogenesis	2	0.019202398	LRIG1, LRIG3
GO:0051281 ~ positive regulation of release of sequestered calcium ion into cytosol	3	0.027727351	GPER1, BDKRB1, PLCG 1
GO:0006835 ~ dicarboxylic acid transport	2	0.028665722	SLC25A10, SLC1A7
GO:0006811 ~ ion transport	5	0.034623955	CHRNB1, TMEM109, SLC25A10, STEAP1, SLC1A7
GO:0006564 ~ L-serine biosynthetic process	2	0.038038297	PSAT1, SHMT2
GO:0055069 ~ zinc ion homeostasis	2	0.047320986	SLC30A4, ATP13A2

Combined with the above overall analysis results, we found that gastric cancer cells in gastric cancer tissues are very insensitive to oxidative stress, and they will not die due to oxidative stress. However, the cells in the microenvironment are extremely sensitive to oxidative stress and easily lead to cell death, especially the T cells (*P* = 1.17E−08). The above results indicate that the oxidative stress on immune cells is likely to be a way for tumors to escape the attack of the immune system. In addition, we also found that mucous cells gradually become cancerous in the microenvironment of gastric cancer, suggesting that the origin of gastric cancer may start from mucous cells.

### Characteristics of potential tumor stem cells in gastric cancer

In the previous related analysis, we found that there are a group of potential cancer stem cells in the single cells derived from gastric cancer tissues, which specifically express ALDH1A2 and EPCAM. Therefore, we hope to understand the characteristics of such potential gastric cancer stem cells.

We used the R package "Seurat" to obtain the differential expression markers between this cell population and the rest of the cell population. We screened low-expressed genes in gastric cancer stem cells, and explored how tumor stem cells inhibit what biological functions. Using the DAVID tool, we performed functional annotations of the top 200 low-expressed genes. We found that this group of cells inhibited "negative regulation of epithelial to mesenchymal transition" and "positive regulation of release of sequestered calcium ion into cytosol" and other functions.

**Fig. 8:**
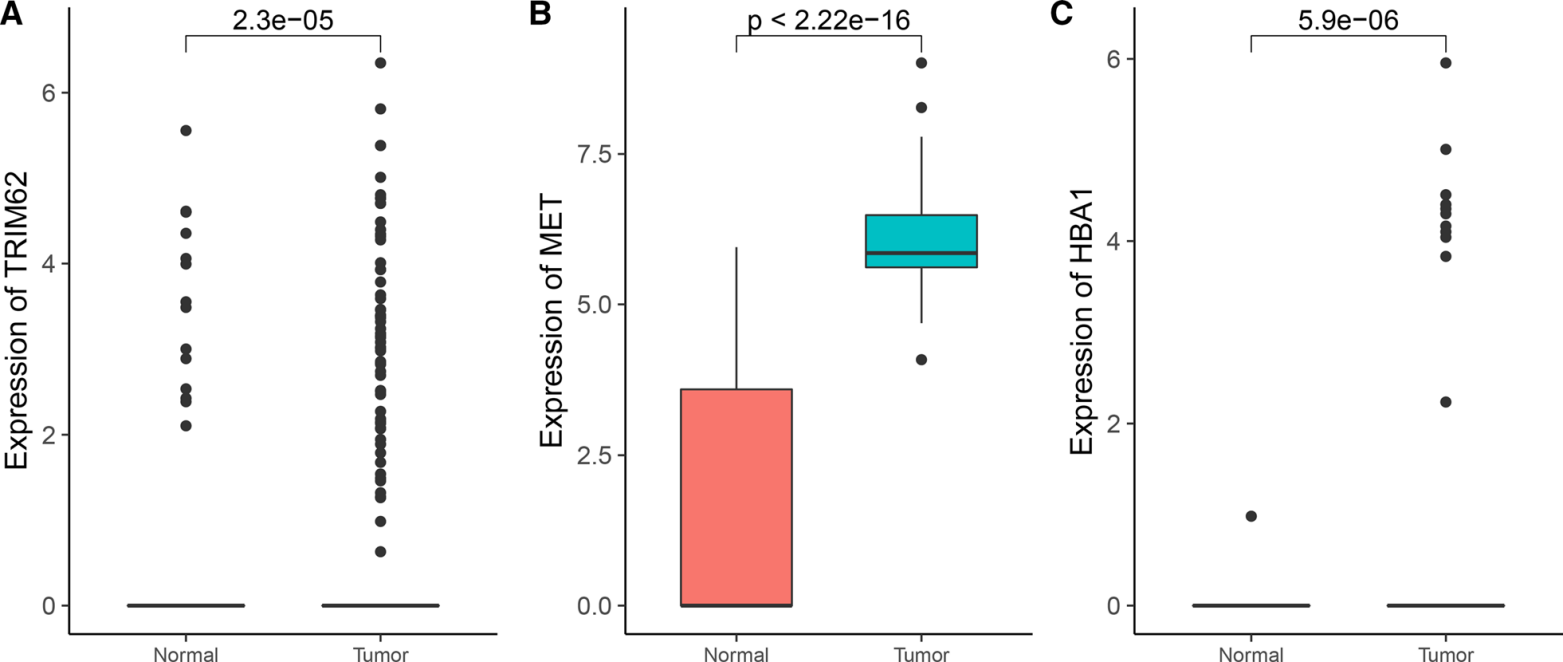
**A** The single cell expression profile of TRIM62 in gastric cancer and normal tissue. **B** The single cell expression profile of MET in gastric cancer and normal tissue. **C** The single cell expression profile of HBA1 in gastric cancer and normal tissue

**Fig. 9:**
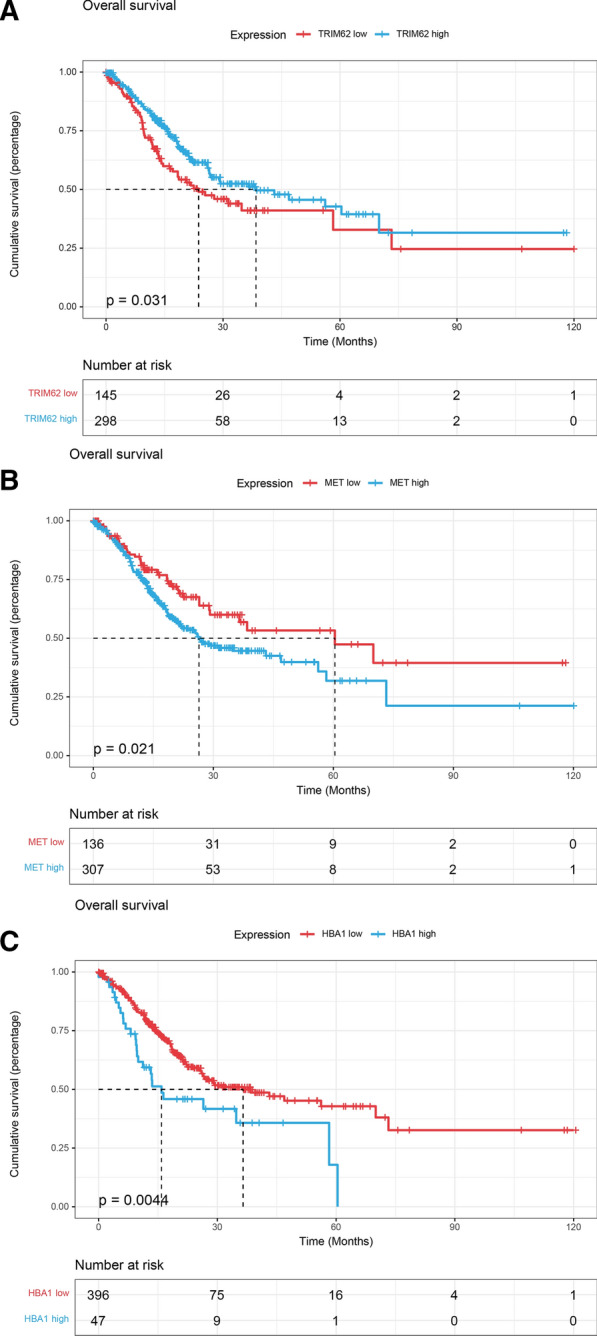
**A** Kaplan–Meier plot of overall survival of TRIM62. **B** Kaplan–Meier plot of overall survival of MET. **C** Kaplan–Meier plot of overall survival of HBA1

Among them, epithelial-mesenchymal transition is a major feature of tumors. This group of cancer stem cells significantly inhibited the three genes TRIM62, HPN, and PBLD that negatively regulate this process. To this end, we conducted a prognostic analysis of these three genes. We obtained 450 samples from gastric cancer patients in the TCGA project that detected gene expression and recorded detailed prognostic information. Based on the expression of these three genes, we conducted prognostic analysis. We found that single cell expression of TRIM62 was increased in tumor samples (Fig. 8A), which was a protective factor for patients with gastric cancer. According to the expression of TRIM62, patients with gastric cancer are divided into high expression group (expression higher than 43.3413) and low expression group (expression lower than 43.3413). The constructed Cox proportional hazard model had HR [90% CI] = 0.7067 [0.5144–0.9709], the log-rank test *P* value was 0.031 (Fig. 9A). However, the expression of HPN and PBLD does not significantly affect patient survival.

### Features of cancerous mucous cell

In addition to cancer stem cells, we also found in the previous analysis that mucous cells derived from tumor tissues gradually began to become cancerous, getting closer and closer to cancer stem cells, and exposed to a high degree of reactive oxygen stress. However, the response to oxidative stress was significantly reduced.

Therefore, we performed differential expression analysis of mucous cells derived from gastric cancer and normal gastric tissue, and extracted genes related to oxidative stress. According to the "GO_RESPONSE_TO_OXIDATIVE_STRESS" oxidative stress response related genes recorded in MsigDB, we obtained a total of 366 related genes, of which 141 genes were differentially expressed, including 22 up-regulated genes and 119 down-regulated genes (Additional file 1: Table S1).

Among them, we found that MET was highly expressed in single cells of tumor samples (Fig. 8B) and cancerous mucous cells, and it has been reported in the literature that it is a proto-oncogene, but there are few reports in gastric cancer. According to the expression value of MET, patients were divided into high expression group (expression higher than 84.25) and low expression group (expression lower than 84.25). The constructed Cox proportional hazard model had HR [95% CI] = 1.512[1.061–2.155], log-rank test *P* value was 0.021 (Fig. 9B).

In addition, we also found that HBA1 was highly expressed in single cells of tumor samples (Fig. 8C) and cancerous mucous cells, and the results of survival analysis show that it is indeed a risk factor. According to the expression value of HBA1, patients were divided into high expression group (expression higher than 68.7978) and low expression group (expression lower than 68.7978), and the constructed Cox proportional hazard model had HR (95% CI) = 1.808(1.196–2.733), log-rank test *P*-value was 0.0044 (Fig. 9C).

## Disccusion

This study systematically clarified the difference in the ssGSEA scores of gastric cancer tissue levels and the microenvironment of gastric cancer in single-cell oxidative stress response, oxidative detoxification, and oxidative stress-induced cell death. The results showed that compared with normal gastric tissue, gastric cancer tissue has a lower oxidative stress response, weaker oxidative detoxification ability, and increased oxidative stress-induced cell death; in the microenvironment of gastric cancer, specific to different types of single cells, we found that the level of T cell oxidative stress response is increased, the ability of oxidative detoxification is enhanced, and the oxidative stress-induced cell death is enhanced. It indicates that the oxidative stress on immune cells is likely to be a way for gastric cancer to evade the immune system. At the same time, in the comparative analysis results of gastric cancer microenvironmental single cells and normal gastric tissues, we can see that Mucous cells show the same trend as gastric cancer cells: poor oxidative stress response, weak oxidative detoxification ability, and weakened oxidative stress-induced cell death, shows that Mucous cell may be a gradually cancerous cell.

Oxidative stress refers to a state in which the oxidation and antioxidant effects in the body are out of balance. Oxidative stress and the resulting oxidative damage are important factors in the occurrence and development of tumors [11, 12]. Studies have compared the oxidative stress levels of the tumor center, tumor margins and healthy tissues of head and neck cancer, and found that the oxidative stress level at the tumor margins was significantly higher than that in the tumor center and healthy tissues, indicating that oxidative stress is related to the occurrence of head and neck cancer [13]. Reuter, er al. summarized the correlation between oxidative stress, inflammation and cancer and points out that oxidative stress can activate transcription factors such as NF-κB, AP-1, p53, HIF-1α, PPAR-γ, β-catenin/Wnt and Nrf2. The activation of these transcription factors can trigger the expression of more than 500 different genes including inflammatory factors. This is closely related to the transformation, survival and proliferation of normal cells to tumor cells [14]. Reactive oxygen species (ROS) is a metabolic by-product produced in the gastrointestinal tract. It is the main attribution of oxidative stress. The oxidative stress caused by it plays an important role in the pathogenesis of gastrointestinal mucosal diseases including gastrointestinal cancer [15]. So far, the pathogenesis of ROS mediated including the occurrence and development of gastrointestinal mucosal diseases is not clear. Huang et al. concluded that ROS participates in the occurrence of gastric cancer by regulating the expression of microRNAs [16]. ROS can not only participate in the process of many diseases by regulating the expression of microRNAs, but it can also participate in the process of long non-coding RNAs (lncRNAs) [17].

The regulation of oxidative stress plays an important role in tumor development and the response to anti-cancer treatments. Gorrini et al. believed that targeted regulation of the antioxidant capacity of tumor cells can produce positive therapeutic effects [18]. In this study, it was found that the ssGSEA score of oxidative stress-induced cell death in gastric cancer tissue was higher than that of normal gastric tissue. Interesting results were found in the microenvironment of gastric cancer. The ssGSEA scores of gastric cancer cells and mucous cells were lower than those of other cell subgroups. Therefore, regulating gastric cancer cells to oxidative stress-induced cell death may have a positive therapeutic effect. In addition, the regulation of oxidative stress response and oxidative detoxification of gastric cancer cells may also produce positive therapeutic effects. For cancer treatment, targeting the tumor microenvironment may become a new strategy for cancer treatment in the future in response to changes in the level of oxidative stress caused by genetic alterations in tumor cells [19].

The microenvironment of gastric cancer is very complex, it contains a variety of cell types: inflammatory cells, fibroblasts, nerve cells and vascular endothelial cells, etc. [5]. Zeng, et al. comprehensively described all the characteristics of the gastric cancer microenvironment and made a detailed analysis of immune infiltration [20]. This study revealed seven cell subgroups in the microenvironmental ecology of gastric cancer through single-cell sequencing data. They are intestinal mucous cells, T cells, Chief cells_1, Chief cells_2, Mucous cells_1, Mucous cells_2 and Tumor cells. It contains a potential gastric cancer cell (ALDH1A2 + and EPCAM +) completely derived from gastric cancer tissue, and ALDH1A2 is a marker of ovarian cancer cancer stem cell [21, 22]. This group of cells inhibited functions such as "negative regulation of epithelial to mesenchymal transition" and inhibited the three genes TRIM62, HPN, and PBLD in the process. After prognostic analysis of the three, TRIM62 was found to be a protective factor for the development of gastric cancer. TRIM62 is a plasmosin, which refers to the E3 ubiquitin ligase related to the RING finger domain. It can catalyze autoubiquitination in vivo and in vitro, and inhibit the proliferation, migration, invasion and Warburg Effect of lung cancer cells in a hypoxic environment [23, 24].

We analyzed the oxidative stress response of different cell subgroups in the microenvironment of gastric cancer and found: increased T cell oxidative stress response level, enhanced oxidative detoxification ability and enhanced oxidative stress-induced cell death, which indicate that the oxidative stress on immune cells is likely to be a way for gastric cancer to escape the immune system. “How can tumors evade immune surveillance and resist immune attacks” has attracted widespread attention [25]. Studies have shown that regulatory T cells are recruited into the human tumor microenvironment and inhibit TAA-specific T cell immunity [26]. It accelerates apoptosis due to its high vulnerability to free oxygen species and weak NRF2-related antioxidant system in the metabolically abnormal tumor microenvironment [27]. This study reveals the possible mechanism of gastric cancer cells evading immune surveillance and resisting immune attack from the single-cell level, which is related to the difference in the oxidative stress level of single cells in the microenvironment of gastric cancer.

In addition, we also found that Mucuous cells and tumor cells in the microenvironment of gastric cancer have the same ssGSEA score trend in the immune stress response, oxidative detoxification ability and oxidative stress-induced cell death. It may be a gradually cancerous cell. Similar results were found in previous single-cell sequencing studies. Glandular mucous cells tend to acquire intestinal stem cell-like phenotypes during metaplasia [28]. The development of intestinal gastric cancer precedes the appearance of the metaplastic cell lineage in the gastric mucosa. The metaplastic cell lineage is characterized by the secretion of mucus. At first, it provides a protective barrier for epithelial cells, but persistent damage and chronic inflammation can make cell reprogramming and metaplasia pattern cycle permanent, which promotes the occurrence of gastric cancer [29]. The genes MET and HBA1, which were significantly related to oxidative stress, were extracted from mucous cells, and their expression significantly affected the survival and prognosis of gastric cancer patients. The results of previous studies have shown that high expression of MET gene predicts a poor prognosis of gastric cancer, which is consistent with our findings [30, 31]. Hemoglobin Subunit Alpha 1 (HBA1) has not been reported much in previous clinical studies on tumor prognosis. Previous bioinformatics analysis showed that it is a key gene in castration-resistant prostate cancer [32]. Our analysis showed that it was also a key gene for predicting the survival and prognosis of gastric cancer.

The present results had pointed out three key genes for the prognosis of gastric cancer. The results of previous bioinformatics analysis identified many key genes associated with the pathogenesis and prognosis of gastric cancer: COL1A1, CXCL8, COL3A1, SPP1, COL1A2, TIMP1, CXCL1, BGN, MMP3 and SERPINE1 [33]. These results are useful for providing a promising therapeutic target in the treatment of cancers [34–36].

## Conclusion

In summary, we analyzed the oxidative stress response of the gastric cancer microenvironment through single-cell sequencing. In the gastric cancer microenvironment, we identified mucous cells with metaplastic characteristics, and speculated that gastric cancer cells evade immune system attacks might be associated with aggravation of T cells death through oxidative stress in the gastric cancer microenvironment. Our analysis also provides potential biomarkers for prognostic survival analysis of gastric cancer related to oxidative stress: TRIM62, MET, and HBA1.

## Supplementary Information

**Additional file 1: Table S1.** Showed the differentially expressed oxidative stress response-related genes. It obtained a total of 366 related genes, of which 141 genes were differentially expressed, including 22 up-regulated genes and 119 down-regulated genes.

## Data Availability

Raw data of this study are available from the corresponding author ZLH upon request.
