# Supplementary material for: Single-cell sequencing analysis reveals gastric cancer microenvironment cells respond vastly different to oxidative stress
Source: J Transl Med. 2022 Jun 3;20:250. doi: 10.1186/s12967-022-03411-w (PMC9164398; doi:10.1186/s12967-022-03411-w)
Supplement: Supplementary file 1 — Additional file 1: Table S1. Showed the differentially expressed oxidative stress response-related genes. It obtained a total of 366 related genes, of which 141 genes were differentially expressed, including 22 up-regulated genes and 119 down-regulated genes. [file 12967_2022_3411_MOESM1_ESM.docx]

Suppl. Table 1. The differentially expressed oxidative stress response related genes

| gene | mean expression in tumor | mean expression in normal | FC | wilcox.test pValue | FDR |
| --- | --- | --- | --- | --- | --- |
| MB | 14.16212766 | 0 | Inf | 1.05E-06 | 9.26E-06 |
| HBA1 | 23.1 | 0.017578947 | 1314.071856 | 5.92E-06 | 4.15E-05 |
| MET | 735.6897872 | 31.85221053 | 23.09697742 | 6.84E-22 | 7.31E-19 |
| HSPB1 | 2297.42 | 242.7584211 | 9.46381176 | 4.85E-21 | 3.75E-18 |
| HBA2 | 38.83978723 | 4.662421053 | 8.330390326 | 0.000104768 | 0.000488367 |
| RHOB | 570.1476596 | 99.15105263 | 5.750293562 | 1.56E-13 | 1.21E-11 |
| PRKCD | 375.7610638 | 73.27778947 | 5.12789846 | 0.001075243 | 0.003527904 |
| JUN | 1515.224681 | 333.0694737 | 4.549275153 | 2.67E-10 | 7.62E-09 |
| HSPA1A | 390.2612766 | 98.458 | 3.963733537 | 9.33E-12 | 4.11E-10 |
| TXNIP | 949.3210638 | 242.3681053 | 3.916856398 | 7.31E-09 | 1.26E-07 |
| DUSP1 | 534.0338298 | 148.3089474 | 3.60082004 | 1.60E-08 | 2.47E-07 |
| HSPA1B | 247.9419149 | 77.86115789 | 3.184410836 | 2.66E-06 | 2.08E-05 |
| KLF2 | 684.9225532 | 228.6466316 | 2.995550595 | 1.54E-06 | 1.30E-05 |
| SESN1 | 45.1493617 | 19.43010526 | 2.323680757 | 0.000149325 | 0.000656246 |
| FOS | 2143.453191 | 1138.718947 | 1.882337337 | 4.27E-05 | 0.00022524 |
| TPM1 | 683.8187234 | 447.2936842 | 1.528791368 | 0.000779986 | 0.002681262 |
| EGFR | 25.49148936 | 19.256 | 1.323820594 | 0.002383943 | 0.006949419 |
| OXSR1 | 39.8512766 | 35.35294737 | 1.127240571 | 0.000503282 | 0.001843555 |
| KPNA4 | 73.25787234 | 69.85178947 | 1.048761569 | 0.001175836 | 0.003794385 |
| FBXO7 | 39.52404255 | 38.906 | 1.015885533 | 0.000878969 | 0.00297157 |
| CDK2 | 14.70404255 | 14.522 | 1.012535639 | 0.000515271 | 0.001880535 |
| NDUFA12 | 203.9078723 | 202.8648421 | 1.005141503 | 6.47E-07 | 6.05E-06 |
| STAU1 | 107.9308511 | 110.0341053 | 0.980885434 | 0.001942825 | 0.005846134 |
| ZNF622 | 30.75957447 | 32.25073684 | 0.953763463 | 0.001248198 | 0.004000044 |
| TXNRD1 | 71.12531915 | 75.30326316 | 0.94451842 | 4.54E-05 | 0.000238178 |
| SETX | 65.42510638 | 74.64515789 | 0.876481586 | 0.002873913 | 0.008163791 |
| NUDT2 | 24.18808511 | 27.60042105 | 0.876366526 | 4.59E-05 | 0.000240166 |
| AKT1 | 73.73276596 | 86.57178947 | 0.851695066 | 1.09E-05 | 7.11E-05 |
| LONP1 | 72.65297872 | 85.83010526 | 0.846474305 | 2.54E-05 | 0.000146122 |
| PYCR2 | 35.02659574 | 42.07747368 | 0.832431053 | 5.35E-05 | 0.000275763 |
| UBQLN1 | 58.08765957 | 70.004 | 0.829776292 | 0.001001127 | 0.003322357 |
| AIFM1 | 42.08382979 | 51.48115789 | 0.817460825 | 0.001528677 | 0.004756764 |
| BNIP3 | 10.49319149 | 12.96894737 | 0.809101247 | 0.003435129 | 0.009519064 |
| XRCC1 | 8.381276596 | 10.45547368 | 0.801616153 | 0.000834753 | 0.002841429 |
| SFPQ | 135.4476596 | 174.7436842 | 0.775121917 | 5.38E-05 | 0.000276737 |
| HYAL1 | 19.49531915 | 26.61021053 | 0.732625513 | 0.000253469 | 0.001031457 |
| ADNP2 | 22.76276596 | 31.15157895 | 0.730709862 | 2.47E-05 | 0.0001432 |
| TRA2B | 112.4612766 | 155.0533684 | 0.725306891 | 1.70E-06 | 1.41E-05 |
| PLEKHA1 | 40.01808511 | 55.67821053 | 0.718738708 | 0.000906475 | 0.003054164 |
| TRAF2 | 10.65978723 | 14.83536842 | 0.71853876 | 0.000303932 | 0.001204038 |
| PPP2CB | 52.68808511 | 77.30484211 | 0.681562547 | 1.60E-06 | 1.35E-05 |
| ARL6IP5 | 69.5693617 | 102.9503158 | 0.675756662 | 8.40E-07 | 7.61E-06 |
| XPA | 36.83744681 | 54.62505263 | 0.674369086 | 0.000509184 | 0.001861253 |
| HMOX2 | 82.8 | 123.5744211 | 0.670041577 | 1.64E-07 | 1.85E-06 |
| BAG5 | 36.18340426 | 54.82473684 | 0.659983182 | 1.41E-06 | 1.20E-05 |
| ETFDH | 22.05787234 | 33.46642105 | 0.659104608 | 0.000316386 | 0.001246306 |
| CYCS | 283.8734043 | 445.4373684 | 0.6372914 | 5.09E-06 | 3.66E-05 |
| IDH1 | 78.87829787 | 124.1793684 | 0.635196481 | 0.001491027 | 0.004655903 |
| PPIF | 108.8693617 | 171.4598947 | 0.634955258 | 0.000513862 | 0.001876378 |
| PRDX5 | 285.3491489 | 457.4749474 | 0.623748143 | 5.51E-07 | 5.26E-06 |
| FBXW7 | 26.8993617 | 43.34652632 | 0.620565567 | 0.000111439 | 0.000515317 |
| PRDX6 | 108.306383 | 175.9632632 | 0.615505652 | 1.01E-08 | 1.66E-07 |
| MDM2 | 29.06914894 | 47.88221053 | 0.607097054 | 1.26E-06 | 1.09E-05 |
| CAT | 42.71702128 | 70.85757895 | 0.602857477 | 4.59E-07 | 4.51E-06 |
| STX4 | 17.5487234 | 29.33915789 | 0.598133166 | 0.000450359 | 0.001679786 |
| MTF1 | 16.67723404 | 28.06073684 | 0.594326305 | 0.000422793 | 0.001591915 |
| HDAC2 | 62.82 | 108.0398947 | 0.581451881 | 6.31E-07 | 5.92E-06 |
| ADAM9 | 97.73787234 | 168.4205263 | 0.580320431 | 5.55E-05 | 0.000284621 |
| GSTP1 | 1095.21 | 1895.334316 | 0.577845286 | 5.05E-07 | 4.91E-06 |
| PDLIM1 | 76.15191489 | 132.8921053 | 0.573035657 | 0.000267237 | 0.001076459 |
| CASP3 | 40.88085106 | 73.75126316 | 0.554307131 | 4.78E-05 | 0.000248707 |
| SOD1 | 176.2804255 | 327.8045263 | 0.537760804 | 2.71E-06 | 2.12E-05 |
| EIF2S1 | 50.99361702 | 95.55210526 | 0.533673401 | 7.61E-07 | 6.98E-06 |
| ATP2A2 | 72.67255319 | 137.2890526 | 0.529339753 | 1.77E-06 | 1.46E-05 |
| APTX | 17.86638298 | 34.04536842 | 0.524781602 | 3.21E-05 | 0.000177705 |
| SLC25A24 | 53.26148936 | 104.904 | 0.507716478 | 5.61E-07 | 5.36E-06 |
| GPX4 | 77.83148936 | 153.7468421 | 0.506231467 | 2.09E-06 | 1.70E-05 |
| EEF2 | 154.146383 | 304.5794737 | 0.506095769 | 9.09E-07 | 8.14E-06 |
| HNRNPM | 116.3751064 | 231.5616842 | 0.502566333 | 1.22E-06 | 1.06E-05 |
| DHFR | 36.26085106 | 73.09210526 | 0.496098052 | 1.83E-05 | 0.000110462 |
| NQO1 | 584.8195745 | 1229.756737 | 0.475557122 | 3.31E-07 | 3.42E-06 |
| TXN | 518.3119149 | 1102.061789 | 0.47031112 | 1.53E-09 | 3.35E-08 |
| NDUFB4 | 114.257234 | 247.1183158 | 0.46235842 | 2.44E-08 | 3.57E-07 |
| RELA | 19.99914894 | 44.21915789 | 0.452273401 | 0.000417881 | 0.001577262 |
| NDUFS2 | 43.90595745 | 97.63463158 | 0.449696555 | 1.39E-06 | 1.18E-05 |
| UBE3A | 38.59787234 | 88.12884211 | 0.437970946 | 1.95E-06 | 1.60E-05 |
| MAPK1 | 39.77914894 | 92.34768421 | 0.430754158 | 3.26E-09 | 6.40E-08 |
| MAPKAP1 | 9.943404255 | 23.35852632 | 0.425686283 | 0.001553019 | 0.004814877 |
| TXN2 | 84.4 | 198.3551579 | 0.425499397 | 1.65E-09 | 3.57E-08 |
| GCLC | 24.62659574 | 58.36347368 | 0.421952194 | 1.24E-05 | 7.93E-05 |
| TRAP1 | 23.30510638 | 58.78726316 | 0.396431219 | 2.82E-08 | 4.05E-07 |
| MSRA | 5.232765957 | 13.26389474 | 0.394512024 | 0.00100435 | 0.003331463 |
| RWDD1 | 60.84212766 | 165.6512632 | 0.367290454 | 4.80E-08 | 6.33E-07 |
| PRDX3 | 43.59808511 | 118.7868421 | 0.367027899 | 8.16E-09 | 1.39E-07 |
| PON2 | 15.57340426 | 42.51431579 | 0.366309653 | 0.000371863 | 0.001436934 |
| PSIP1 | 19.00723404 | 52.20284211 | 0.364103433 | 4.15E-06 | 3.06E-05 |
| P4HB | 376.6078723 | 1038.574421 | 0.362620015 | 4.63E-13 | 3.01E-11 |
| ABL1 | 9.704468085 | 28.34757895 | 0.342338515 | 0.000288865 | 0.001151567 |
| HNRNPD | 89.18340426 | 269.3885263 | 0.331058659 | 1.96E-10 | 5.81E-09 |
| SRXN1 | 9.356595745 | 28.84073684 | 0.324422909 | 8.40E-07 | 7.61E-06 |
| HSPD1 | 113.2580851 | 350.8708421 | 0.322791385 | 2.14E-07 | 2.32E-06 |
| RPS3 | 732.3797872 | 2303.491474 | 0.317943346 | 1.50E-15 | 2.02E-13 |
| LDHA | 114.356383 | 364.7756842 | 0.313497823 | 4.26E-11 | 1.54E-09 |
| DHCR24 | 36.35404255 | 116.4157895 | 0.312277593 | 4.90E-09 | 9.08E-08 |
| PRDX1 | 187.6085106 | 605.6269474 | 0.309775698 | 7.95E-12 | 3.55E-10 |
| MSRB2 | 14.66702128 | 47.90652632 | 0.306159148 | 0.001232051 | 0.00395468 |
| AKR1C3 | 202.5538298 | 672.798 | 0.301061879 | 2.41E-12 | 1.26E-10 |
| TP53 | 17.17170213 | 57.30957895 | 0.299630576 | 1.56E-07 | 1.78E-06 |
| ATRN | 6.912765957 | 23.142 | 0.298710827 | 7.09E-05 | 0.000351241 |
| EZH2 | 16.87851064 | 58.58673684 | 0.288094397 | 1.27E-06 | 1.10E-05 |
| PNPT1 | 11.59297872 | 41.932 | 0.276470923 | 5.24E-06 | 3.75E-05 |
| TMEM161A | 5.104255319 | 19.12494737 | 0.266889901 | 0.003001573 | 0.008471052 |
| FUT8 | 19.65574468 | 73.91442105 | 0.265925707 | 3.35E-07 | 3.45E-06 |
| NONO | 34.37468085 | 134.1472632 | 0.25624586 | 7.42E-10 | 1.80E-08 |
| PRDX2 | 95.27808511 | 380.0155789 | 0.250721524 | 1.32E-12 | 7.46E-11 |
| PRKRA | 10.70595745 | 42.99989474 | 0.248976364 | 9.94E-05 | 0.0004674 |
| SGK2 | 3.501702128 | 14.13715789 | 0.247694915 | 0.00240458 | 0.007000772 |
| PKD2 | 1.456808511 | 5.966210526 | 0.244176518 | 0.002565584 | 0.007407484 |
| PYCR1 | 35.15808511 | 149.2309474 | 0.235595134 | 4.34E-11 | 1.56E-09 |
| GLRX2 | 11.7693617 | 54.03115789 | 0.217825458 | 2.92E-06 | 2.27E-05 |
| NDUFS8 | 65.78106383 | 308.8576842 | 0.212981795 | 5.79E-12 | 2.70E-10 |
| PRNP | 10.32808511 | 49.05105263 | 0.210557869 | 1.05E-06 | 9.26E-06 |
| PRDX4 | 41.47531915 | 207.1301053 | 0.200238005 | 1.91E-13 | 1.44E-11 |
| REST | 39.8812766 | 200.3186316 | 0.199089202 | 0.000250795 | 0.001022971 |
| GCLM | 5.856170213 | 29.64284211 | 0.19755765 | 0.000126098 | 0.000570575 |
| ALDH3B1 | 9.457446809 | 51.81315789 | 0.182529828 | 1.90E-08 | 2.87E-07 |
| MPV17 | 5.638723404 | 32.91505263 | 0.17131139 | 6.00E-07 | 5.66E-06 |
| NDUFA6 | 58.62382979 | 344.8025263 | 0.170021462 | 8.60E-13 | 5.25E-11 |
| PARP1 | 27.59787234 | 164.7105263 | 0.167553786 | 2.18E-10 | 6.35E-09 |
| MGST1 | 44.78638298 | 268.5997895 | 0.166740201 | 9.10E-14 | 7.71E-12 |
| PPP5C | 4.118723404 | 25.40105263 | 0.162147745 | 1.66E-07 | 1.87E-06 |
| PCNA | 39.29680851 | 248.2428421 | 0.158299865 | 8.50E-10 | 2.00E-08 |
| PSMB5 | 22.74297872 | 144.2318947 | 0.157683422 | 1.14E-10 | 3.61E-09 |
| FANCD2 | 3.745957447 | 24.33726316 | 0.153918599 | 0.000692401 | 0.002428205 |
| IMPACT | 5.458297872 | 37.03284211 | 0.147390736 | 1.85E-05 | 0.000111408 |
| GPX1 | 35.10340426 | 255.4129474 | 0.137437842 | 3.54E-13 | 2.45E-11 |
| PARK7 | 36.0687234 | 307.6923158 | 0.117223348 | 8.95E-18 | 2.74E-15 |
| ATG7 | 0.856595745 | 7.394210526 | 0.115846816 | 0.003264416 | 0.009092889 |
| FYN | 1.65893617 | 15.604 | 0.106314802 | 0.000295138 | 0.001172877 |
| SIGMAR1 | 8.420638298 | 80.12810526 | 0.105089697 | 3.13E-11 | 1.17E-09 |
| SIRT1 | 1.428510638 | 15.98073684 | 0.089389535 | 0.002485777 | 0.007196488 |
| MTR | 3.152553191 | 36.77589474 | 0.085723358 | 5.21E-09 | 9.58E-08 |
| APEX1 | 11.19106383 | 136.4021053 | 0.082044656 | 6.95E-14 | 6.16E-12 |
| NUDT15 | 5.81106383 | 71.37147368 | 0.081419978 | 6.23E-11 | 2.14E-09 |
| AIFM2 | 2.743617021 | 50.12894737 | 0.054731192 | 2.14E-08 | 3.17E-07 |
| FXN | 2.245531915 | 41.40389474 | 0.054234799 | 6.79E-11 | 2.31E-09 |
| G6PD | 0.889574468 | 16.82463158 | 0.05287334 | 0.000494154 | 0.001815858 |
| ADA | 0 | 8.074105263 | 0 | 5.97E-05 | 0.000301996 |
| PPARGC1B | 0 | 18.86515789 | 0 | 5.63E-06 | 3.97E-05 |
| ETV5 | 0 | 7.050526316 | 0 | 0.000376622 | 0.001447464 |
| OGG1 | 0 | 7.589684211 | 0 | 0.001084098 | 0.003540251 |
